# Time has come to provide infertile men with an optimal fertility pathway

**DOI:** 10.1590/S1677-5538.IBJU.2019.0362.1

**Published:** 2021-03-25

**Authors:** Sandro C. Esteves

**Affiliations:** 1 Centro de Referência para Reprodução Masculina Clínica de Andrologia e Reprodução Humana CampinasSP Brasil ANDROFERT, Clínica de Andrologia e Reprodução Humana, Centro de Referência para Reprodução Masculina, Campinas, SP, Brasil; 2 Universidade Estadual de Campinas - UNICAMP Departamento de Cirurgia (Disciplina de Urologia) CampinasSP Brasil Departamento de Cirurgia (Disciplina de Urologia), Universidade Estadual de Campinas - UNICAMP, Campinas, SP, Brasil; 3 Aarhus University Faculty of Health Aarhus Denmark Faculty of Health, Aarhus University, Aarhus, Denmark

## COMMENT

Intracytoplasmic sperm injection (ICSI) has become the most common method of fertilization used in assisted reproductive technology (ART), accounting for over 85% of ART cases in South America (1). Among many driving forces explaining the widespread use of ICSI, the possibility of its use in virtually all causes of infertility is foremost. This has led to a dismissal of the need to evaluate the male partner and/or improve his sperm quality (2-4).

However, the worldwide delivery rates with ICSI remains low (∼30%-40%), and data accumulated over the last 25 years suggest that congenital malformations, epigenetic disorders, chromosomal abnormalities, subfertility, cancer, delayed psychological and neurological development, and impaired cardiometabolic profile are increased in infants born as a result of ICSI compared with naturally conceived children (5). Therefore, several professional societies have advised against the indiscriminate use of ICSI.

As ICSI experience increased, it became evident that the paternal factor plays a role in its success rates (4, 6). Moreover, reports suggesting that interventions aimed at ameliorating sperm quality before ICSI could increase its success rates are increasing steadily (7). Nonetheless, a comprehensive evaluation is seldom performed on men attending Fertility Clinics. A further impediment to providing a full male investigation is the relative lack of reproductive urologists on fertility clinic teams. Gynecologists staff most fertility clinics, so they are female-focused. Traditionally, these clinicians have little training in the causes of male infertility and hence in the male partner. This requires urgent redress as a matter of best clinical practice.

In a recent article, Pariz and colleagues illustrate the importance of a comprehensive clinical and laboratory investigation of men seeking fertility (8). They studied an exemplary young couple who had undergone two failed ICSI cycles with the husband's sperm. Notably, the primary ICSI indication was based on the results of a single semen analysis that revealed complete asthenozoospermia (100% immotile sperm). The female partner was apparently normal, and the male partner had not undergone any urologic evaluation despite showing a severe sperm abnormality. After ICSI, no oocyte fertilized on both occasions, prompting the couple to seek a second expert opinion.

Using a comprehensive workup, Pariz et al. found an asthmatic male with situs inversus, who had unremarkable findings on clinical examination despite that. Nevertheless, the laboratory investigation was much more elusive, confirming not only the picture of complete asthenozoospermia but also revealing complete teratozoospermia (100% abnormal sperm morphology). Also noteworthy was the finding that most sperm were alive on a viability staining test. The laboratory investigation included a search for infection, a prudent step to take in such cases, which was negative. Additionally, a panel of sperm functional tests was utilized to assess oxidative stress markers and sperm DNA fragmentation (SDF). Of interest, lack of mitochondria activity was detected on sperm midpiece, whereas increased SDF levels were noticed by assessing the neat ejaculate using the sperm chromatin structure assay. Lastly, electron microscopy (EM) revealed remarkable defects on sperm centrosome and tail, typical of those found in men with primary ciliary dyskinesia (PCD) syndrome. Of note, sperm centrioles were significantly affected.

The authors are commended for conducting such an elegant investigation and add to our understanding of this complex condition. Moreover, the case highlights the importance of a proper male evaluation, which is paramount, particularly when an abnormal semen analysis is found. As highlighted by several guidelines, the infertile man should be evaluated by a urologist with expertise in infertility. While the routine semen analysis remains the cornerstone of laboratory investigation (9), and it is usually the reason for the referral, infertility due to a paternal factor may also occur in the absence of evident abnormalities in the routine semen analysis (7). Sperm functional tests, mainly those that assess sperm chromatin integrity, have become essential tools to investigate the role of a possible paternal factor and guide clinical management. In these lines, readers are invited to examine recently published articles in this and other Journals, highlighting the clinical utility of including sperm functional tests to the male infertility work-up (2, 4, 6, 7, 10-12).

In the case discussed by Pariz and co-workers, the comprehensive laboratory investigation was critical to confirm the PCD diagnosis and unravel a poor sperm quality that likely explained the previous ICSI failures. The authors also added to the literature by suggesting that sperm redox balance seems to be well-preserved in PCD men. Thus, oxidative stress (OS) –often regarded as the main causative factor in sperm DNA fragmentation (6)– cannot explain the elevated SDF levels seen in the studied patient. Interestingly, the EM showed disrupted sperm nuclear condensation that likely explains the SDF findings.

Based on the authors’ observations and the possible association between SDF and ICSI fertilization failure, it might be speculated that SDF testing would be of clinical value in men with PCD, a hypothesis in need of further confirmation. Notwithstanding the possible association between SDF and fertilization, the central element explaining the complete fertilization failure after ICSI in the studied case seems to relate mainly to a combination of factors involving axonemal, nuclear, and mitochondria alterations.

**Figure 1:**
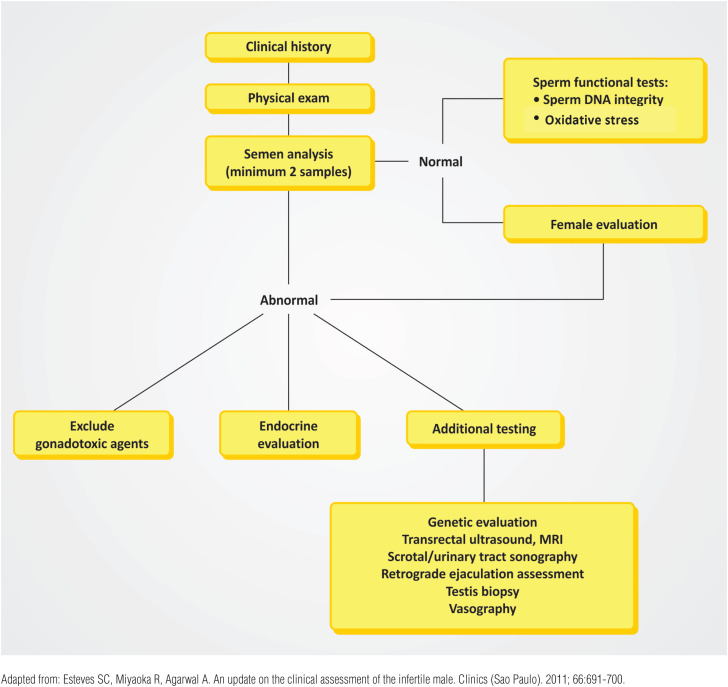
Simplified Algorithm for the Initial Male Infertility Evaluation.

This exemplary case also highlights that ART may not bypass the most severe sperm defects. Although ICSI has been a tremendous achievement for helping couples achieve biological parenthood, it is our opinion that the method should not be overused. Its indication in the context of infertility must be made after both partners are adequately evaluated (13-15). For guidance, a simplified male infertility workup algorithm is depicted in Figure-1.

The male infertility evaluation goals are not only to identify conditions that can be corrected or those that are irreversible for which ART will be needed. Also important is identifying serious medical issues that may be causing or associated with male infertility and require medical treatment. In these lines, efforts should be made to identify genetic causes of male infertility that may affect the success of treatment or the health of offspring if ART is utilized (16, 17). Lastly, as in the report of Pariz et al., it is also critical to identify irreversible conditions for which the male partner's sperm will not be available or appropriate, thus requiring consideration of donor sperm or adoption.
